# Flavonolignans Inhibit IL1-β-Induced Cross-Talk between Blood Platelets and Leukocytes

**DOI:** 10.3390/nu9091022

**Published:** 2017-09-15

**Authors:** Michal Bijak, Angela Dziedzic, Ewelina Synowiec, Tomasz Sliwinski, Joanna Saluk-Bijak

**Affiliations:** 1 Department of General Biochemistry, Faculty of Biology and Environmental Protection, University of Lodz, Pomorska 141/143, 90-236 Lodz, Poland; angela.dziedzic@outlook.com (A.D.); joanna.saluk@biol.uni.lodz.pl (J.S.-B.); 2 Laboratory Medical Genetics, Faculty of Biology and Environmental Protection, University of Lodz, Pomorska 141/143, 90-236 Lodz, Poland; ewelina.synowiec@biol.uni.lodz.pl (E.S.); tomasz.sliwinski@biol.uni.lodz.pl (T.S.)

**Keywords:** interleukin 1, anti-inflammatory, flavonolignans, silybin, silychristin

## Abstract

Interleukin-1 beta (IL-1β)—the most potent pro-inflammatory is responsible for a broad spectrum of immune and inflammatory responses, it induces T-cell and B-cell activation and consequently the synthesis of other pro-inflammatory cytokines (such as IFN-γ and TNF). IL-1β induces the formation of blood platelet-leukocyte aggregates (PLAs), which suggests that IL-1β significantly affects the cross-talk between blood platelets and the immune response system, leading to coronary thrombosis. The aim of our study is to investigate the effect of flavonolignans (silybin, silychristin and silydianin) on the IL-1β-induced interaction between platelets and leukocytes, as well as on the expression and the secretion of pro-inflammatory factors. Whole blood samples were pre-incubated with commercially available flavonolignans (silybin, silychristin and silydianin) in a concentration range of 10–100 µM (30 min, 37 °C). Next, samples were activated by IL-1β for 1 h. Blood platelet-leukocyte aggregates were detected by using the double-labeled flow cytometry (CD61/CD45). The level of produced cytokines was estimated via the ELISA immunoenzymatic method. IFN-γ and TNF gene expression was evaluated using Real Time PCR with TaqMan arrays. We observed that in a dose-dependent manner, silybin and silychristin inhibit the IL-1β-induced formation of blood platelet-leukocyte aggregates in whole blood samples, as well as the production of pro-inflammatory cytokines—IL-2, TNF, INF-α, and INF-γ. Additionally, these two flavonolignans abolished the IL-1β-induced expression of mRNA for IFN-γ and TNF. Our current results demonstrate that flavonolignans can be novel compounds used in the prevention of cardiovascular diseases with dual-use action as antiplatelet and anti-inflammatory agents.

## 1. Introduction

Interleukin 1 beta (IL-1β) is the most potent pro-inflammatory cytokine that is crucial in host-defense responses to infection and injury [1]. IL-1β is expressed by many cells and has multiple functions, including in local inflammation. IL-1β is produced by activated macrophages, endothelial cells, B cells, and fibroblasts. This potent pro-inflammatory cytokine was initially discovered and classified as the major endogenous pyrogen. IL-1β mediates the expression of a vast array of genes involved in secondary inflammation, which coordinate all aspects of local inflammation and also attract and activate the cells of the adaptive immune system at the infection sites [2]. IL-1β is responsible for a broad spectrum of immune and inflammatory responses, induces T-cell and B-cell activation, and consequently the synthesis of other pro-inflammatory cytokines (such as IFN-γ, IL-6 and TNF), and antibody production. This cytokine also induces the expression of itself in newly-arriving monocytes, thus reinforcing the overall process. IL-1β circulating in blood is unregulated under systemic and chronic inflammatory conditions and is measurable in pg/mL [3].

The mechanism of IL-1β cell action is based on the binding to type I IL-1 receptor (IL-1RI) and the activation of the intracellular signal pathway. IL-1β first binds to the first extracellular chain of IL-1RI that recruits the IL-1 receptor accessory protein (IL-1RAcP), which serves as a co-receptor and is necessary for signal transduction. In response to the ligand binding of the receptor, a complex sequence of combinatorial phosphorylation and ubiquitination events results in the activation of nuclear factor κB (NF-κB) signalling and the JNK and p38 mitogen-activated protein kinase pathways. Together, these then induce the expression of canonical *IL-1β* target genes through transcriptional and post-transcriptional mechanisms [4].

Pro-inflammatory cytokines and chemokines can affect all of the coagulation pathways. Therefore, the relationship between the presence of cytokines resulting in inflammation and hyper-coagulation state, is particularly relevant in the pathogenesis of thrombosis. Interleukin 1 Receptor 1 and IL-1β have been seen to be increased in cardiovascular diseases [5]. IL-1β is also known to be present in autoimmune conditions and contributes to several chronic diseases, including atherosclerosis [6]. Increased levels of IL-1β are known to play an important role in both acute and chronic inflammation, with resulting pathological clotting. However, there is still little information available about the effects of this interleukin on the properties of blood platelet involved in clot formation. An in vitro study performed using the flow cytometry method indicated that IL-1β significantly increases the formation of blood platelet-leukocyte aggregates (PLAs). This suggests that IL-1β significantly effects the cross-talk between blood platelets and the immune response system [5]. Flavonolignans are a group of active chemical components of silymarin—an extract obtained from the fruit of the milk thistle—*Silybum marianum* (L.) Gaernt. [7]. This plant, which is a member of Asteraceae family, has been used for thousands of years as a remedy for a variety of ailments [8]. Flavonolignans are structurally composed of a flavonoid unit (taxifolin) and a phenylpropanoid unit (coniferyl alcohol), linked by an oxeran ring [9,10]. This type of connection is present in the formation of lignans, and gives this group of compounds its name [11]. Silymarin represents 1.5–3% of the dry fruit weight. The main represents of flavonolignans presented in silymarin are silybin, isosilybin, silychristin, isosilychristin silydianin, silimonin [7,12,13,14,15,16], however the highest concentration, approximately 70% of the extract have the silybin, silychristin and silydianin and these compounds are the major bioactive component of extract [17]. In our previous study, we demonstrated that flavonolignans, especially silybin and silychristin, are able to adenosine diphosphate (ADP)-induce blood platelets’ activation through interactions with the P2Y12 receptor [18]. Additionally, silybin and silychristin have an inhibitory effect on platelets cyclooxygenase activity, which blocks arachidonic acid metabolism in these cells [19].

Recent studies demonstrate that the flavonolignans are able to inhibit the NF-κB activation pathway, which is responsible for cell reaction to IL-1β. For this reason, we decided to investigate the effect that flavonolignans (silybin, silychristin and silydianin) have on the IL-1β-induced interaction between platelets and leukocytes, as well as on the expression and secretion of pro-inflammatory and prothrombotic factors.

## 2. Materials and Methods

### 2.1. Reagents

Interleukin-1 beta was purchased from Miltenyi Biotec (Bergisch Gladbach, Germany). Dimethyl sulfoxide (DMSO), Tris and the flavonolignans (silybin, silychristin and silydianin (Figure S1) were all obtained from the Sigma-Aldrich Chemical Co. (St. Louis, MO, USA). Flow cytometry reagents: anti-CD61/FITC, anti-CD61/PE, anti-CD45/PE, isotype controls, BD FACS^TM^ Lysing Solution and CellFix were all obtained from Becton Dickinson (San Diego, CA, USA). All of the other chemicals were of reagent grade or the highest quality available.

### 2.2. Blood Samples

Blood samples collected from twelve different healthy donors were purchased from the Regional Centre for Transfusion Medicine in Lodz (Poland). All of the samples had been drawn in the morning (between 8 a.m. and 10 a.m.), from fasting donors and immediately transferred to the laboratory. All donors had been checked by a doctor and were found to have had no cardiovascular disorders, allergies, lipid, or carbohydrate metabolism disorders, nor any traces of medication. Blood was collected according to the standard protocol to the CPDA-1 (Citrate Phosphate Dextrose Adenine Solution) containing blood collection bag with double port, 450 mL (KRUUSE, Langeskov, Denmark). Our analysis of the blood samples was performed under the guidelines of the Helsinki Declaration for Human Research, and approved by the Committee on the Ethics of Research in Human Experimentation at the University of Lodz (with Resolution No. 16/KBBN-UŁ/II/2016).

### 2.3. Samples Preparation

The fresh whole blood samples were pre-incubated with flavonolignans (silybin, silychristin and silydianin) in the concentration range of 10–100 µM, at 37 °C. All of the compounds tested were initially dissolved in 20% DMSO to a preliminary concentration of 20 mM. Other solutions of the compounds used were also 20% DMSO (prepared in 50 mM Tris-buffered saline [TBS], pH 7.4). The final DMSO concentration of all the samples was 0.1%. In the control samples, the same volume of solvent was added (20% DMSO prepared at 50 mM TBS, pH 7.4), with the probes warmed at 37 °C [18,19,20]. After 30 min, to each sample (control or pre-incubated with flavonolignans) IL-1β (10 ng/mL) was added. Treatment with IL-1β was conducted for 1 h at 37 °C, and samples were used for appropriate analysis. An additional sample was not activated.

### 2.4. Flow Cytometry Analysis of Platelet-Leukocyte Aggregates

First, the blood samples were stained in BD FACS lysing solution. After 1 h of fixation, the samples were stained with specific antibodies: anti-CD61/FITC, anti-CD45/PE (6 µL of each antibody + 50 µL of sample), and left for 30 min in the dark, at room temperature. Next, 500 µL of 1% Cellfix was added to each sample. All of the samples were centrifuged (2500× *g*, 10 min), and the precipitate obtained was then suspended in 500 µL of 0.9% NaCl. The fluorescence of 10,000 leucocytes (CD45/PE-positive objects) was measured using the CUBE 6 (Pertec, Görlitz, Germany) flow cytometer. Blood platelet-leukocyte aggregates were detected using CD61-FITC and CD45-PE fluorescence (Figure S2). The specific fluorescence fractions were obtained after the subtraction of nonspecific fluorescence in the control samples (labelled with proper isotype control). Gates for PE and FITC fluorescents were estimated based on the fluorescence of unstained probes. The percentage values of CD61+/CD45+ positive objects (PLAs) were calculated relative to the total number of leucocytes (CD45 positive cells) present in each sample. All of the data analysis was performed in CyFlow version 1.5.1.2 (Pertec, Görlitz, Germany).

### 2.5. Cytokine Level Analysis

After preparation, the samples were centrifuged (2500× *g*, 15 min) to obtain plasma. The following cytokine levels: Interleukin 2 (IL-2), tumuor necrosis factor (TNF), interferon α (INF-α), interferon γ (INF-γ), transforming growth factor β (TGF-β), were all measured using commercial ELISA kits (Mabtech, Nacka Strand, Sweden) in accordance with the manufacturer’s protocol. All of the measurements were made using MaxiSorp plates (Nunv, Roskilde, Denmark). Absorbance was measured at 450 nm using the SPECTROstar Nano Microplate Reader (BMG Labtech, Ortenberg, Germany).

### 2.6. Isolation of RNA and Reverse Transcription

Frozen whole blood samples (−80 °C) were lysed using TRI Reagent^®^ (Sigma-Aldrich), after which separation was performed. Then the InviTrap Spin Universal RNA Mini Kit (Stratec Biomedical Systems, Birkenfeld, Germany) was used to purify the RNA-containing aqueous phase. The quantity and purity of RNA were estimated using a Synergy HTX Multi-Mode Microplate Reader equipped with a Take3 Micro-Volume Plate (BioTek Instruments, Inc., Winooski, VT, USA). Total RNA (0.15 µg) was reverse transcribed into cDNA with a High-Capacity cDNA Reverse Transcription Kit (Applied Biosystems™, Waltham, MA, USA). All of the steps were performed according to the manufacturer’s recommendations.

### 2.7. Real-Time PCR

Expression levels of both studied genes were obtained using the following TaqMan probes: Hs00174128_m1 for the human *TNF* gene*,* Hs00989291_m1 for the human *INF-γ* gene, and Hs99999901_s1 as an endogenous control, which was the human *18S rRNA* gene (Life Technologies, Carlsbad, CA, USA). Real-time PCR analyses were performed using a CFX96 real-time PCR system (BioRad Laboratories, Hercules, CA, USA) with a TaqMan Universal Master Mix II, without UNG (Life Technologies). All procedures were performed according to the manufacturers’ protocols. Relative expressions of the studied genes were calculated using the equation 2^−ΔCt^, where ΔCt = Ct_target gene_ − Ct_18*S rRNA*_. 

### 2.8. Statistical Analysis

All experiments were performed in duplicate, calculated as mean values and expressed as mean ± SD. All-statistical analyses were performed using Stats Direct statistical software Version. 2.7.2 (StatsDirect software, Cheshire, UK). The results obtained were analysed for normality using a Shapiro-Wilk test. Next, the results were analysed for equality of variance using Levene’s test. The significance of the differences between the values was analysed using ANOVA, followed by Tukey’s range test for multiple comparisons (for data with normal distribution and equality of variance), and the Kruskal-Wallis test; *p* < 0.05 was accepted as statistically significant.

## 3. Results

### 3.1. Flavonolignans Effect on IL-1β-Induced Formation of Blood Platelet-Leukocyte Aggregates

**Figure 1:**
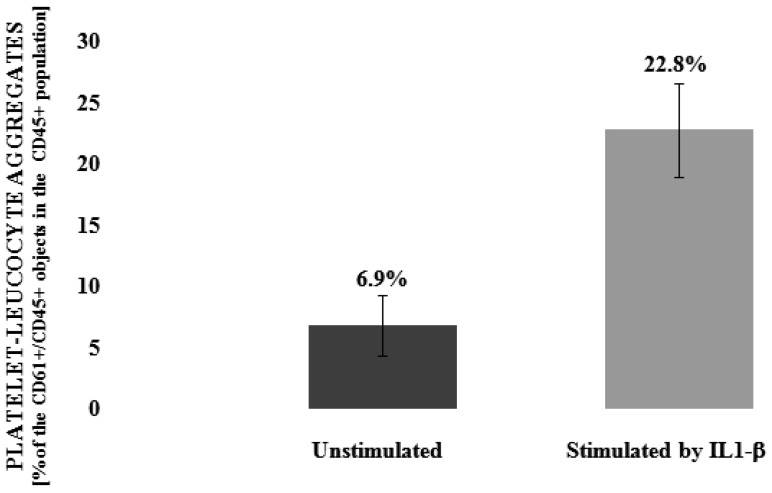
The effect of IL-1β (10 ng/mL) on the formation of blood platelet-leukocyte aggregates. Results of double-label flow cytometry measurements are expressed as the amount of CD61+/CD45+ objects in the whole CD45+ population (presented as %), *n* = 12, *p* < 0.001.

**Figure 2:**
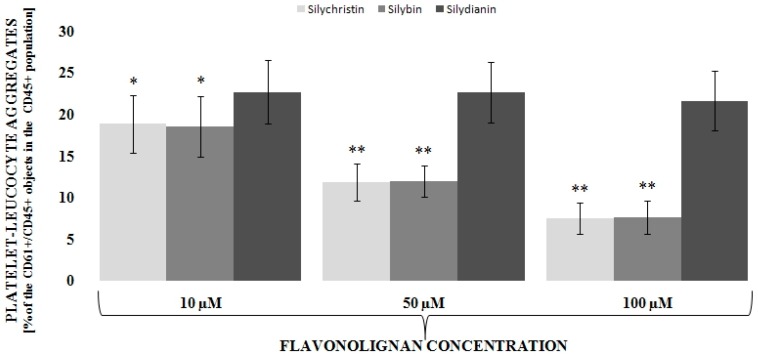
The effect flavonolignans; silychristin, silybin and silydianin in concentrations of 10, 50, and 100 µM on the IL-1β (10 ng/mL) induced the formation of blood platelet-leukocyte aggregates. The results of double-label flow cytometry measurements are expressed as the amount of CD61+/CD45+ objects in the whole CD45+ population (presented as %), *n* = 12; * *p* < 0.01, ** *p* < 0.001.

To determine the level of interaction between blood platelets and leucocytes we applied double-label flow cytometry as an investigative method. Based on flow cytometry measurements, our results clearly indicate that IL-1β is statistically significant (*p* < 0.001) in the induction (about three times–6.9% vs. 22.8%) of the formation of platelets-leukocytes aggregates (Figure 1). Next, we observed that, dose dependent, two of the three tested flavonolignans–silychristin and silybin–inhibit the IL-1β-induced formation of blood platelet-leukocyte aggregates in the whole blood samples (Figure 2). In the highest used concentration (100 µM), it was observed that both silychristin and silybin are able to reduce the formation of platelet-leukocyte aggregate formation in IL-1β-induced samples to similar values, as observed in the control samples (without IL-1β)—22.8% vs. 7.5% and 7.6%, respectively.

### 3.2. Flavonolignans Effect on IL-1β-Induced Cytokine Production (IL-2, TGF-β, TNF, INF-α and INF-γ)

In the next step, we determined the effects of flavonolignans on IL-1β-induced cytokine production by blood cells using the ELISA method. For this analysis, we selected 5 cytokines: IL-2, TGF-β, TNF, INF-α, and INF-γ. In all of them, except for TGF-β, after IL-1β treatments of blood samples a statistically significant increase was observed. The highest induction of production by IL-1β was observed for TNF (about 5 times—from 355 pg/mL to 1632 pg/mL), and INF-γ (about 7 times—from 255 pg/mL to 1701 pg/mL). Subsequently, we evaluated the inhibitory effect of flavonolignans on the IL-1β-induced production of cytokines by blood cells. In all samples in which the blood had been treated with silybin and silychristin, a reduction of cytokine concentration was observed (Table 1). In samples treated with 100 µM silychristin and 100 µM silybin, the levels of produced cytokines were reduced by about 90%. In samples treated with silydianin, we observed some inhibitory tendencies, however, none were statistically significant.

### 3.3. Silychristin and Silybin Effect on mRNA Expression for INF-γ and TNF Genes

**Figure 3:**
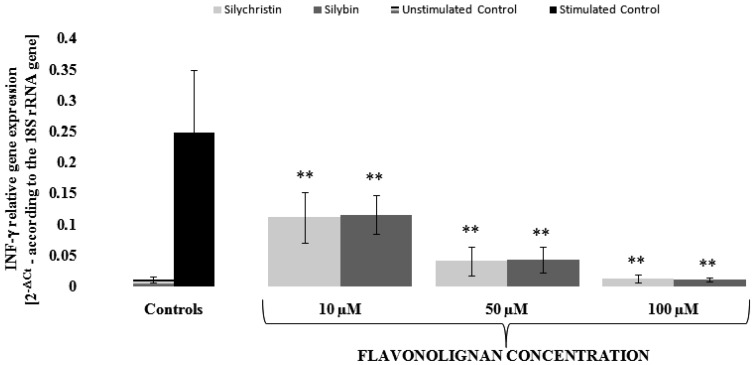
The effect of flavonolignans; silychristin and silybin in concentrations of 10, 50, and 100 µM on the IL-1β (10 ng/mL) induced the expression of *INF-γ* gene (measured at the mRNA level). The results are expressed as a mean of 2^−∆ct^ (according to the reference gene-*18S rRNA*) ± SD, *n* = 12; ** *p* < 0.001.

**Figure 4:**
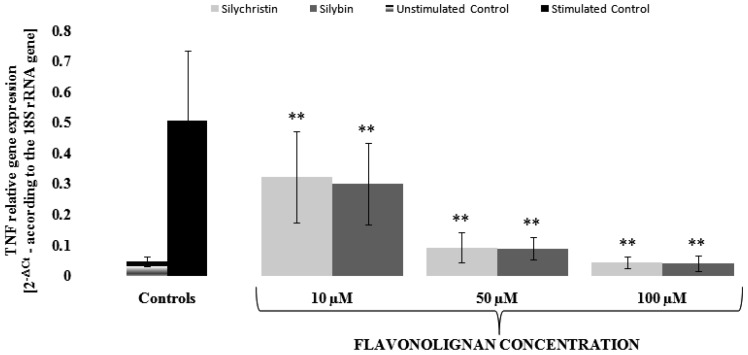
The effect of flavonolignans; silychristin and silybin in concentrations of 10, 50, and 100 µM on the IL-1β (10 ng/mL) induced the expression of *TNF* gene (measured at the mRNA level). The results are expressed as a mean of 2^−∆ct^ (according to the reference gene—18*S rRNA*) ± SD, *n* = 12; ** *p* < 0.001.

In order to evaluate the mechanism of silychristin and silybin anti-inflammatory effects, we performed a gene expression analysis at the mRNA level using the Real-Time PCR method. We decided to evaluate changes in mRNA expression for INF-γ and TNF genes, for which expression was induced by IL-1β (about 25 [0.0101 vs. 0.2473] and 11 [0.0462 vs. 0.508] times, respectively). In our measurements, we found that, in a dose-dependent manner, both silychristin and silybin inhibit the IL-1β-induced expression of INF-γ and TNF genes at the mRNA level (Figure 3 and Figure 4). Both compounds have similar inhibitory effects, with the strongest observed at the highest concentration (100 µM), in which gene expressions were reduced to a level observed in the control samples without IL-1β treatment. 

## 4. Discussion

Thrombosis and inflammation are closely related pathophysiological processes with multicellular activation involving blood platelets and leukocytes. Coronary artery disease, including Acute Coronary Syndromes (ACS), which refers to group of clinical symptoms is compatible with acute myocardial ischaemia, associated with coronary artery thrombosis is one of the most common causes of death in the world. It is now believed that elevated levels of inflammatory factors in the blood promote the development of cardiovascular events, and that chronic inflammation plays a key role in the pathogenesis of atherosclerosis and acute coronary syndromes. Much intensive study by various leading scientific centres around the world confirms that the balance between pro-and anti-inflammatory processes influence the risk of developing ACS [21]. In blood samples obtained from persons with acute coronary syndrome episodes, elevated concentrations of chemokines (IL-8, MCP-1, eotaxin, MIP-1α, and IP-10) and cytokines (IL-1, IL-6, IL-7, IL-12, IL-17, IFN-α, and granulocyte-macrophage colony-stimulating factor) regulating both innate and adaptive immunity have been observed [22].

Numerous studies form cardiovascular disease have shown that the platelet-leukocyte interaction (so-called crosstalk) was increased [23]. Thrombosis and inflammation involve complex platelet-leukocyte interaction, the details of which have not been fully elucidated. Under conditions that mimic a physiological state, the platelet-leukocyte cross-talk involves multiple mediators and mechanisms [24], and is a common feature of atherothrombosis and inflammatory immune reactions. In the last few years, it has been suggested that platelet-leukocyte interactions contribute to cardiovascular disease [25]. The creation of PLAs involves the recruitment leukocytes to the atherosclerotic plaques and stimulates them to release collagenases such as MMP-8, MMP-9, and proteinase 2, which affect the reduction of the atherosclerotic plaque stability by degrading the collagen of the extracellular matrix [26]. As a result of pathological platelet activation, there is an increased immune response and an increasing number of platelet-leukocyte complexes formed at the site of the atherosclerotic plaque, which can lead to its rupture [27]. An increased number of platelet-leukocyte aggregates circulating in blood have been observed in patients with ACS [28]. Additionally, in patients who died following an ACS episode, platelet-neutrophil interactions occurring at the site of ruptured plaques have been observed [29]. Flow-Cytometric analysis of platelet aggregation showed the significant effect of IL-1β on the formation of PLAs, showed the significant effect of IL-1β on the formation of PLAs, which suggests that IL-1β significantly affects pro-inflammatory and prothrombotic cross-talk between platelets and leukocytes. In the current study, we have also observed the very strong ability of IL-1β to induce the formation of blood platelet-leukocyte aggregates (Figure 1). However, this effect was abolished with the application of two flavonolignans: silychristin and silybin. Depending on the dose, these two inhibit the formation of blood platelet-leukocyte aggregates induced by IL-1β (Figure 2).

Leukocytes enhance the inflammatory process within the atherosclerotic plaque’s formation. Pro-inflammatory cytokines, such as TNF and INF-γ, as inducers of endothelial cell activation and expression of adhesive particles, play a key role in the recruitment of leukocytes, particularly monocytes, from the blood stream [30]. TNF and IFN-γ are particularly toxic to endothelial cells, and activate monocytes, macrophages, and phospholipase A2, which intensifies the synthesis of pro-inflammatory eicosanoids in the atherosclerotic plaque [31]. Additionally, these inflammatory mediators exacerbate the expression of MMPs in macrophages, as well as in endothelial and smooth muscle cells. MMP activity, regulated by inflammatory mediators, is responsible for the disintegration of interstitial collagen, leading to thinning and fibrous cup (FC) depletion, causing a susceptibility to atherosclerotic plaque’s fracture [32]. INF-γ also inhibits the ability of smooth muscle cells to synthesize the new interstitial collagen fibres required for the repair of the FC extracellular matrix [33].

In our study, we observed that of all the tested cytokines (IL-2, TNF, INF-α, INF-γ, TGF-β), IL-1β most induces the production of TNF and INF-γ (Table 1). Additionally, in this study, we have observed that the two tested flavonolignans: silybin and silychristin, reduce the IL-1β-induced production of cytokines. In the highest concentration of the tested compounds (100 µM), we observed the almost complete abolition of the pro-inflammatory action of IL-1β.

Contrary to numerous in vitro and in vivo studies on flavonolignans, including investigations of hepatoprotective activity [34], their anti-inflammatory properties and the therapeutic effects have been less thoroughly described. However, some information is available [35]. The biochemical mechanisms include the modulation of a variety of cell-signalling pathways, resulting in the reduction of pro-inflammatory mediators. Both silymarin [36] and pure silybin [37] suppress NF-κB, which plays a crucial role in regulating immune response and inflammation through the regulation of the expression of various genes involved in these processes [38]. A non-activated NF-κB is maintained in the cytoplasm by the inhibitory protein 1-κBα (IκBα). The activation of NF-κB occurs via the phosphorylation of IκBα, leading to its proteasome-mediated degradation, release from IκBα complex, and the translocation to the nucleus. NF-κB pathway plays an essential role in activating genes encoding pro-inflammatory cytokines (TNF, IFN, IL-1β, IL-2, IL-6, and granulocyte macrophage colony-stimulating factors), chemokines (IL-8, macrophage inflammatory protein 1α, macrophage chemotactic protein 1), enzymes that generate mediators of inflammation (5-lipoxygenase), immune receptors (interleukin-2 receptors), and also adhesion molecules (E-selectin, intercellular adhesion molecule 1) [39]. The study presented by Trappoliete et al. [40], shows that silybin is able to inhibit the IκBα phosphorylation, which suppresses the IL-1β-induced activation of the NF-κB pathway in hepatic stellate cells (HSC). Silybin was also able to suppress the antigen-stimulated calcium uptake and the activation of NF-κB, resulting in the significant reduction of TNF and IL-6 production [41]. In the present study, in samples treated with silybin and silychristin, we observed a reduction of the levels of cytokines secreted into plasma, as well as the inhibition of IL-1β-induced expression of TNF and IFN-γ, which confirms the anti-inflammatory effect of these two flavonolignans. Additionally, in the last few years, novel forms of flavonolignans administration have been developed that posses a very high bioavailability (with plasma concentrations ranging from 60–70 µM) [42]. This corresponds with the concentrations of flavonolignans that have a biological effect in our study (10–100 µM).

## 5. Conclusions

In summary, our results indicate that flavonolignans may be used in the prevention of cardiovascular disease with dual action as antiplatelet and anti-inflammatory agents. Further studies using a larger sample size and additional studies, demonstrating NF-κB activity is necessary before the final statement about the role of the inhibitory effect of these two flavonolignans on the NF-κB pathway.

## Figures and Tables

**Table 1:** The effect of flavonolignans; silychristin, silybin and silydianin in concentrations of 10, 50, and 100 µM on the IL-1β (10 ng/mL) induced the production of pro-inflammatory cytokines (IL-2, TNF, INF-α, INF-γ, TGF-β). The levels of pro-inflammatory cytokines were estimated in plasma obtained from whole blood samples treated with IL-1β and flavonolignans and presented as a mean of concentration ± SD, *n* = 12; * *p* < 0.05, ** *p* < 0.01, *** *p* < 0.001.

Cytokine	Control (without IL1-β)	Control (with IL1-β)	IL1-β + Silychristin (µM)			IL1-β + Silybin (µM)			IL1-β + Silydianin (µM)		
			10	50	100	10	50	100	10	50	100
INF-γ (pg/mL)	255 ± 90	1701 ± 411 ***	1278 ± 339 **	555 ± 175 ***	262 ± 82 ***	1339 ± 337 **	574 ± 149 ***	283 ± 87 ***	1677 ± 398	1665 ± 483	1542 ± 405
TNF (pg/mL)	355 ± 110	1632 ± 473 ***	1216 ± 396 **	478 ± 160 ***	338 ± 119 ***	1286 ± 408 *	466 ± 115 ***	352 ± 115 ***	1614 ± 453	1610 ± 423	1494 ± 360
INF-α (pg/mL)	13.3 ± 6.4	20.8 ± 8.0 **	16.8 ± 6.1	15.0 ± 4.9 *	13.3 ± 4.0 ***	16.3 ± 4.4 *	14.8 ± 4.5 *	12.6 ± 3.5 ***	20.1 ± 5.1	18.8 ± 4.4	17.9 ± 3.9
IL-2 (pg/mL)	116 ± 28	189 ± 49 **	168 ± 45	129 ± 19 **	116 ± 24 ***	164 ± 38	135 ± 21*	112 ± 17 ***	191 ± 46	175 ± 31	172 ± 30
TGF-β (pM)	78.3 ± 23.3	86.8 ± 27.9	84.2 ± 24.1	83.6 ± 28.1	83.6 ± 27.9	86.8 ± 31.8	82.8 ± 19.2	82.3 ± 27.7	84.1 ± 24.5	87.2 ± 23.5	85.3 ± 25.1

## Supplementary Materials

The following are available online at www.mdpi.com/2072-6643/9/9/1022/s1. Figure S1: Chemical structures of flavonolignans used in this study, Figure S2: Flow cytometry dot plot presented blood platelet-leukocyte aggregates.
