# Supplementary material for: Flavonolignans Inhibit IL1-β-Induced Cross-Talk between Blood Platelets and Leukocytes
Source: Nutrients. 2017 Sep 15;9(9):1022. doi: 10.3390/nu9091022 (PMC5622782; doi:10.3390/nu9091022)
Supplement: Supplementary file 1 [file nutrients-09-01022-s001.zip › nutrients-222422-supplementary.pdf]

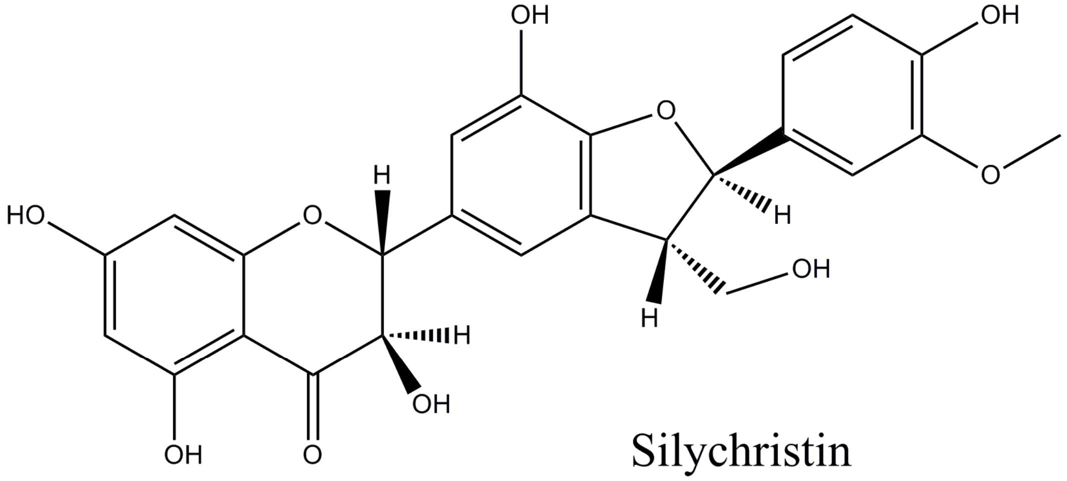

Silychristin

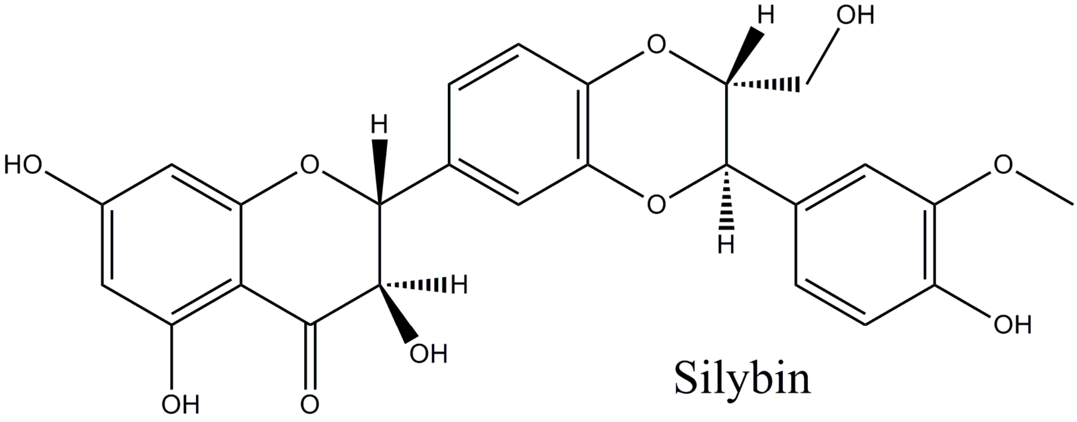

Silybin

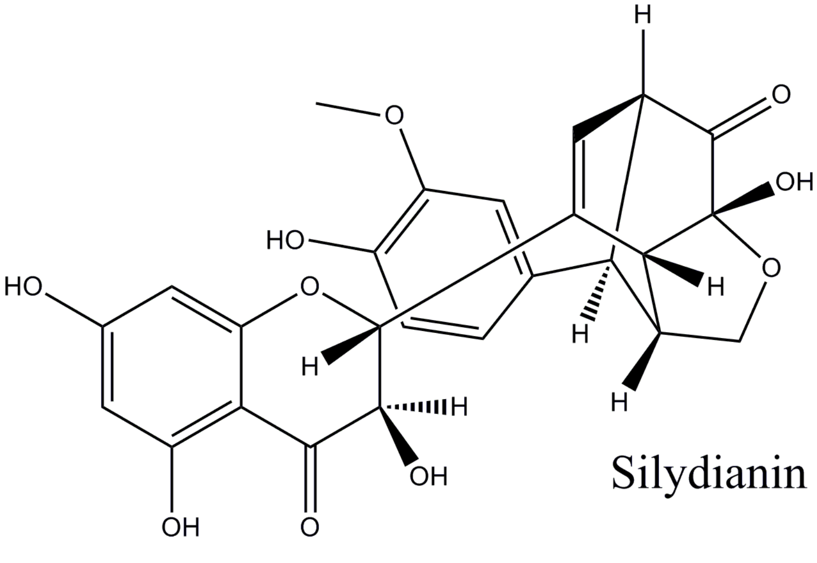

Silydianin

Figure S1: Chemical structures of flavonolignans used in this study

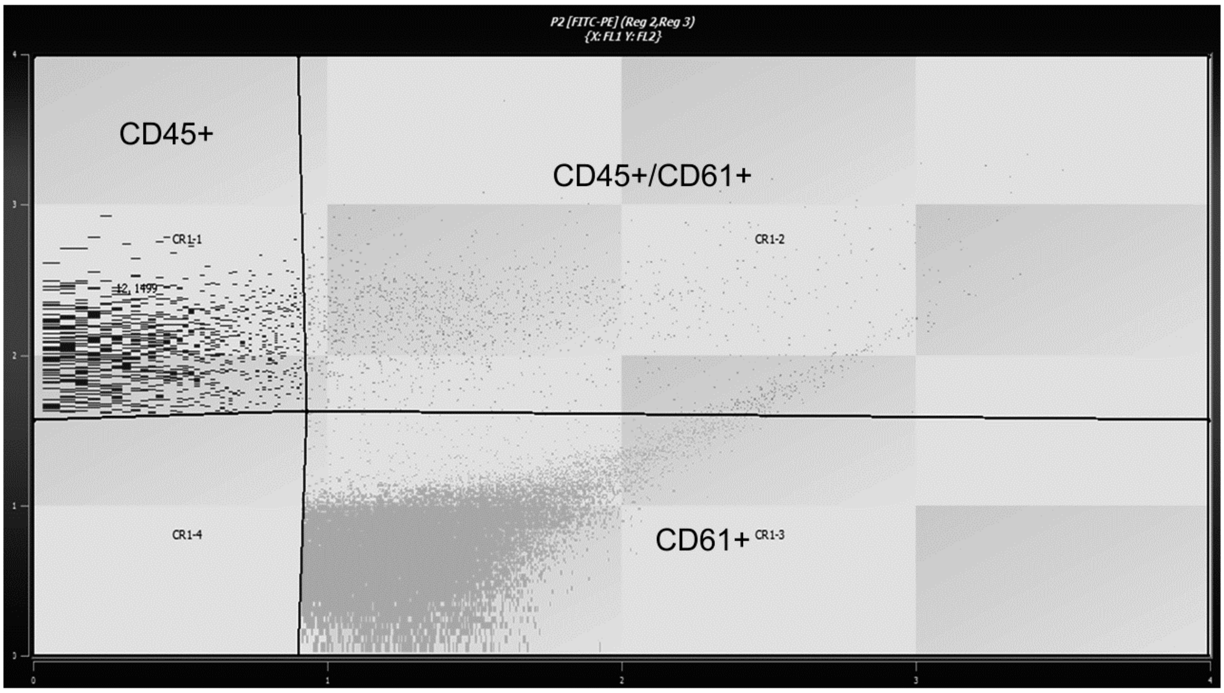

Figure S2: Flow cytometry dot plot presented blood platelet-leukocyte aggregates
